# Calcium dobesilate-induced hyperpyrexia: A case report

**DOI:** 10.1097/MD.0000000000035785

**Published:** 2023-10-27

**Authors:** Hui Yang, Hong-Ling Yuan, Zhi-Ping Zhang, Hong-Kui Zhang, Ming-Wei Liu

**Affiliations:** a Department of Nephrology, The First Affiliated Hospital of Kunming Medical University, Kunming, Yunnan, China; b Department of Emergency, The First Affiliated Hospital of Kunming Medical University, Kunming, Yunnan, China, 650032

**Keywords:** calcium dobesilate, case report, fever, misdiagnosis, treatment

## Abstract

**Rationale:**

Calcium dobesilate, a vasoprotective and antioxidant agent, is gradually being used for the treatment of chronic kidney disease. Calcium dobesilate-induced hyperpyrexia is a rare clinical event, and few studies have reported it.

**Patient concerns:**

The patient took calcium dobesilate, which caused high fever. After stopping calcium dobesilate, his body temperature returned to normal.

**Diagnoses:**

Based on the medical history, symptoms and signs, the patient was diagnosed with drug fever caused by calcium dobesilate.

**Interventions:**

Calcium dobesilate was stopped, and supportive treatment was given at the same time.

**Outcomes:**

The present case was initially misdiagnosed as a fever caused by a bacterial infection, but treatment with the antibiotic moxifloxacin was ineffective. Based on the patient’s medical history, laboratory and examination results, body temperature changes, and Naranjo Advanced Drug Response Scale, calcium dobesilate-induced hyperpyrexia was diagnosed. After discontinuation of calcium dobesilate, the patient’s body temperature normalized, and no additional episode of fever was observed at follow-up.

**Lesson:**

Moreover, misdiagnosis and mistreatment of this condition can deteriorate the patient’s condition. Herein, we report a case of calcium dobesilate-induced hyperpyrexia that occurred during the treatment of chronic renal insufficiency. Subsequently, a systematic analysis of the patient’s diagnosis and treatment was reviewed. If unexplained high fever develops during the use of calcium dobesilate, calcium dobesilate-induced hyperpyrexia should be considered. Accordingly, calcium dobesilate should be discontinued.

## 1. Introduction

Calcium dobesilate, a vasoprotective agent, reduces pathological hyperpermeability by increasing microvascular wall resistance and decreases blood and plasma viscosity and platelet hypercoagulability. This effect is antithrombotic and eliminates or relieves edema, capillary oozing, lower extremity heaviness, and pressure sensation. Therefore, calcium dobesilate is widely used in diabetic retinopathy, varicose vein syndrome, and various microvascular diseases.^[1]^ Renal microvasculature is involved in renal injury and is a bearer of metabolic, inflammatory, and other injury factors. Therefore, renal microangiopathy is a determinant of renal function deterioration.

Calcium dobesilate protects renal microvasculature and improves renal function by effectively inhibiting microvascular hyperpermeability caused by vasoactive drugs and promoting basement membrane collagen synthesis. Calcium dobesilate can improve the treatment outcome and survival of patients with chronic renal insufficiency.^[2,3]^ Calcium dobesilate-induced hyperpyrexia is rare in clinical settings, with little research reported. This report presents a case of calcium dobesilate-induced hyperpyrexia in treating chronic renal insufficiency.

## 2. Case information

### 2.1. Ethics approval

Informed written consent was secured from the legal next of kin for the patient for the publication of this case report and the accompanying images. This study was reviewed and approved by the local ethics committee of the First Affiliated Hospital of Kunming Medical University. The procedures were in accordance with the Helsinki Declaration of 1975, as revised in 2000.

### 2.2. General information

An 80-year-old male patient was admitted to our hospital on December 4, 2021, at 16:54 with a chief complaint of recurrent chills, cold intolerance, and hyperpyrexia for the past 2 days.

### 2.3. Medical history

The patient presented with a chief complaint of chills, cold intolerance, fever with a maximum temperature of 39.0 °C, and generalized aching pains and weakness for the past 2 days without any obvious cause. The symptoms were slightly relieved after taking Sanjiu Ganmaoling granules; however, they recurred soon after. Routine blood and urine tests, coronavirus disease 2019 (COVID-19) nucleic acid tests, and chest computed tomography (CT) imaging performed at Ganmei Hospital of Kunming First People’s Hospital (Kunming, China) 1 day prior were normal, and an initial diagnosis of “the cause of fever is to be investigated (respiratory tract infection? Urinary tract infection?).” After taking oral “ibuprofen and moxifloxacin hydrochloride tablets 0.4 g once a day, and Lianhua Qingwen capsule 1.4 g 3 times a day.” Orally, the patient’s body temperature decreased to 36.5 °C the next morning. The same afternoon, the patient developed chills, cold intolerance, and fever without any obvious cause once again. The patient was referred to our hospital for further treatment, where he was admitted to the emergency department after undergoing emergency COVID-19 nucleic acid testing in the fever clinic. During the course of the disease, the patient had poor mental health, diet, and sleep but normal bowel movements. Moreover, no significant weight change was observed.

### 2.4. Previous history

The patient had a history of hypertension for the past 12 years with a maximum blood pressure of 180/110 mm Hg. The patient routinely took oral “lacidipine 4 mg once every morning and benazepril hydrochloride 5 mg once a night,” which provided good blood pressure control. The patient was diagnosed with chronic renal insufficiency 1 month ago and was prescribed calcium dobesilate 0.5 g po tid, medicinal charcoal tablets 3 po tid, and Sanqi Danshen tablets since November 27, 2021.

### 2.5. Physical examination results

Physical examination performed on admission revealed the following: temperature, 39.8 °C; pulse, 104 beats/minutes; respiratory rate, 20 breaths/minutes; and blood pressure, 129/65 mm Hg. Additionally, the patient was conscious, cooperative on physical examination, and generally in good condition. Physical examination also revealed a noncongested pharynx, normal tonsils, bilateral clear breath sounds, and no significant dry or moist rales. His heart rate was 104 beats per minute. A cardiac examination revealed no abnormal heart sounds or pericardial friction sounds. The patient’s abdomen was flat and soft with no pressure pain, rebound pain, or muscle tension, and no bilateral edema was detected. Furthermore, the patient’s physiological reflexes were normal, and no pathological reflexes were elicited.

### 2.6. Auxiliary examination results

**Figure 1.**
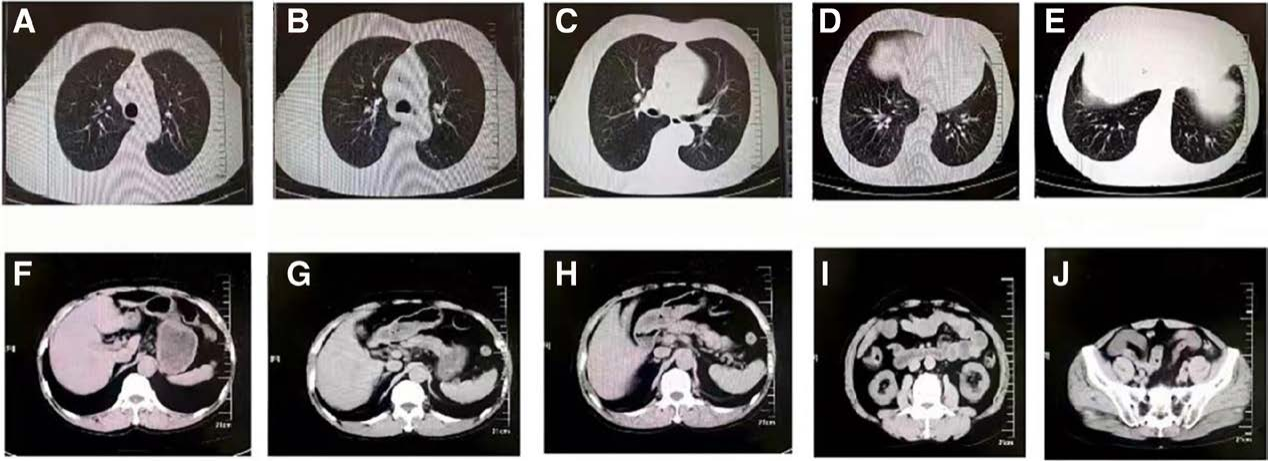
Changes in chest and abdominal computed tomography (CT) at the time of patient admission. (A–E) Changes in the thoracic CT at the time of patient admission. (F–J) Changes in abdominal CT of the patient upon admission. CT = computed tomography.

Routine blood tests performed at our hospital on December 4, 2021, revealed the following: white blood cell level, 5.2 * 10^9/L; neutrophil percentage, 74.2%; procalcitonin level, 0.45 ng/mL; and ultrasensitive C-reactive protein level, 53.4 mg/L. Blood biochemistry results revealed the following levels: sodium, 126 mmol/L; chloride, 98.93 mmol/L; albumin, 34.5 g/L; anhydride, 222.7 µmol/L; amylase, 159 u/L; glucose, 6.7 mmol/L; troponin I, 0.029 ng/mL; myoglobin, 112.18 ug/L; and brain natriuretic peptide, 35.38 pg/mL. Moreover, routine urine tests, electrocardiograms, and emergency coagulation and fibrinolytic tests revealed normal results. A plain CT scan of the abdomen showed the following (Fig. 1): Possible multiple liver cysts; Multiple kidney cysts; A slightly thickened lateral branch of the left adrenal gland; Para-spleen; and Calcified spots in the abdominal aorta and bilateral iliac vessel walls. Chest CT findings were as follows (Fig. 1): Bilateral, multiple lung microscopic nodules with partial calcification; Localized calcified spots in the wall of the thoracic aorta; and Enlarged mediastinal lymph nodes with localized calcification. Blood culture results did not suggest any abnormalities. Moreover, the nucleic acid test for COVID-19 was negative.

### 2.7. Diagnosis and treatment

**Figure 2.**
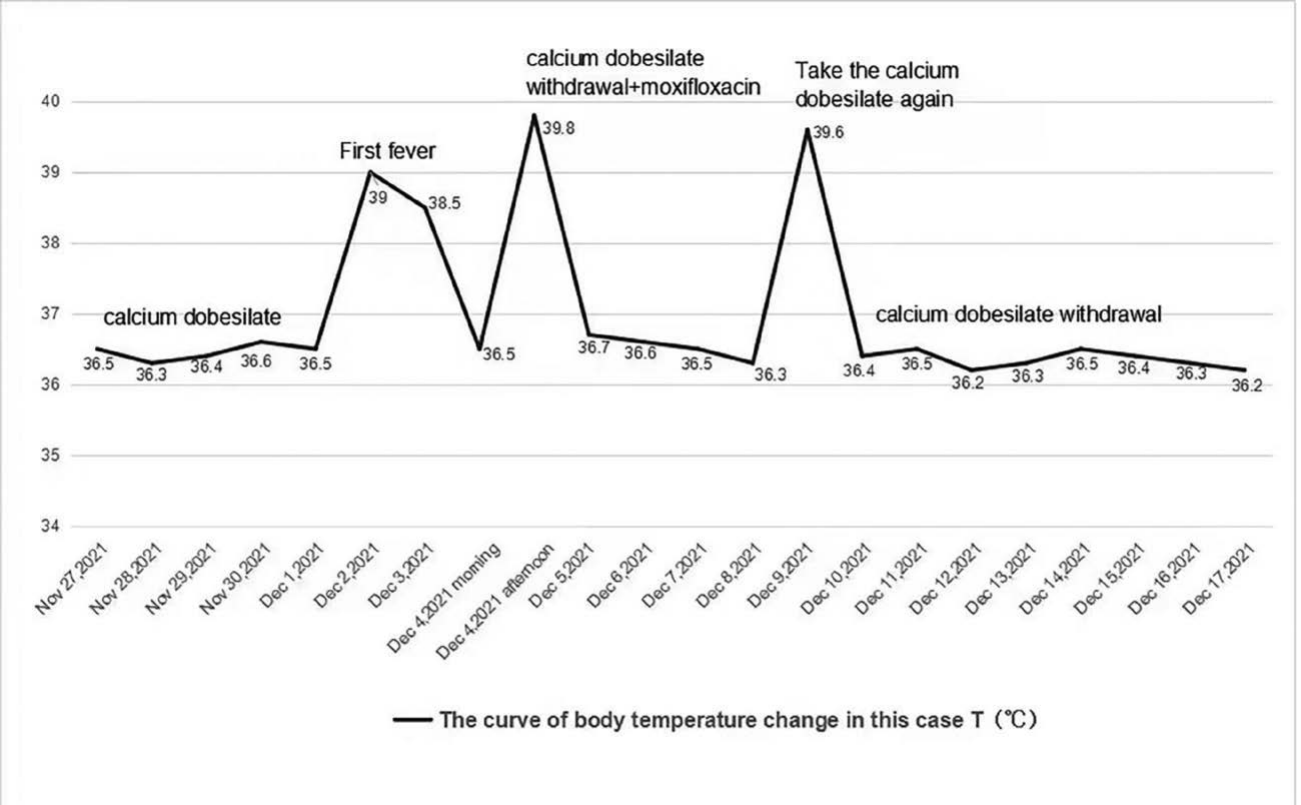
Changes in body temperature of patients before and after taking calcium dobesilate and during follow-up.

According to the patient’s medical history, ancillary examination findings, changes in the patient’s body temperature (Fig. 2) and the rating of the Naranjo Adverse Drug Reactions Scale (Table 1), and possible causes of the fever, including possible infectious fever and drug-induced fever, calcium dobesilate was discontinued on December 4, 2021. Additionally, the patient was administered intravenous moxifloxacin sodium chloride 250 ml for empirical anti-infective treatment and hyponatremia and hypochloremia correction. The patient’s body temperature was 36.7 °C on December 5, 2021, and the patient continued moxifloxacin-based anti-infection and oral rehydration therapy with rehydration salts along with hospitalization for observation. The patient’s temperature on December 6 was 36.6 °C, and he was in good general condition. On December 7, the patient’s temperature was 36.5 °C, and he was in good general condition. Moreover, routine blood tests did not indicate significant abnormalities. Simultaneously, hyponatremia and hypochloremia were also corrected, and the patient recovered well. Therefore, moxifloxacin was discontinued, and the patient was discharged from the hospital for follow-up. On December 9, the patient developed a fever again with a temperature of 39.6 °C. According to the medication history inquiry, the patient developed a fever after continuing calcium dobesilate after discharge from the hospital (December 9), indicating calcium dobesilate-induced hyperpyrexia. Therefore, calcium dobesilate was discontinued, following which the patient’s temperature decreased to 36.4 °C on December 10.

**Table 1:** Naranjo score table of this patient.

Questions	Score			
	Yes	No	Unknown	Score
1. Whether the ADR previously had a conclusive report	1	0	0	1
2. Whether the ADR occurred after the use of suspicious drugs	2	−1	0	2
3. Whether the ADR was relieved after withdrawal or administration of antagonists	1	0	0	1
4. Does the ADR repeat after the reuse of suspicious drugs	2	−1	0	2
5. Is there any other reason that can cause the ADR alone	−1	2	0	−1
6. Does the ADR repeat after placebo	−1	1	0	0
7. Does the drug reach a toxic concentration in blood or other body fluids	1	0	0	0
8. Does the ADR increase with the increase of the dose, or alleviate with the decrease of the dose	1	0	0	0
9. Has the patient ever been exposed to the same or similar drugs and had a similar reaction	1	0	0	1
10. Is there any objective evidence to confirm the reaction	1	0	0	0
Total score	6			

definite: ≥9; probable: 5–8; possible; 1–4; doubtful ≤ 0.

ADR = adverse drug reactions

### 2.8. Follow-up

At the 2-week follow-up, the patient reported no episode of fever after discontinuing calcium dobesilate.

## 3. Discussion

Fever is caused primarily by infectious diseases, noninfectious inflammatory diseases, and tumors, with infectious fever being the most common.^[4–7]^ Drug fever, another cause of fever, refers to fever occurring during drug use and disappearing after discontinuation of the drug. Drug fever is classified as an adverse drug reaction and is rarely seen in clinical practice.^[8,9]^ Therefore, it is often overlooked by clinicians, resulting in delayed diagnosis and treatment.^[10]^ The present case was first misdiagnosed as having infectious fever in another hospital and was administered antibacterial treatment. After discontinuation of calcium dobesilate in our hospital, the patient’s body temperature normalized, and no additional episode of fever was observed at follow-up.

Most antipyretic measures are ineffective in drug fever, and its diagnosis assumes that the cause of the patient’s fever cannot be explained by other etiologies. Particularly, when the fever is incompatible with a possible infection, the cessation of fever after discontinuation of drug administration establishes the initial diagnosis of drug fever. Recurrence of fever after drug readministration confirms the diagnosis of drug fever. The symptoms of patients with drug fever are often not consistent with the degree of fever.^[11]^ In the present case, despite his high body temperature, the patient was in good general condition, which was disproportionate to the high body temperature.

In the present case report, the patient had a score of 6 on the Naranjo Adverse Drug Reactions Scale,^[12]^ which indicated that the fever was probably caused by calcium dobesilate administration. The patient started taking calcium dobesilate on November 27 for chronic renal insufficiency and developed hyperpyrexia, chills, and cold intolerance intermittently on December 2. At the beginning of the drug administration, also called the drug sensitization period, the patient did not present these symptoms. On the day of admission, the patient’s C-reactive protein level was elevated, indicating a normal stress reaction due to persistent hyperpyrexia.^[13]^ The patient developed hyperpyrexia, chills, and cold intolerance after several doses of calcium dobesilate. However, the body temperature decreased to normal after discontinuing the drug. Therefore, the diagnosis of calcium dobesilate-induced hyperpyrexia was definite.

Common side effects of calcium dobesilate include abdominal pain, diarrhea, nausea, vomiting, arthralgia, and myalgia. However, fever is a rare side effect of calcium dobesilate ingestion. Some pharmaceutical manufacturers of calcium dobesilate, such as Calcium Dobesilate Capsules (Tianan Pharmaceutical Co., Ltd, Guizhou, China, 0.25 g/tablet, Approval No.: SFDA Approval Number H20010481), Calcium Dobesilate Dispersible Tablets (Linheng Pharmaceutical Co., Ltd, Hainan, China, 0.25 g/tablet, Approval No.: SFDA Approval Number H20080644), Calcium Dobesilate Capsules (Lijun Pharmaceutical Co., Ltd, Xi’an, China, 0.5 g/tablet, Approval No.: SFDA Approval Number H20000713), and Calcium Dobesilate Tablets (Changao Pharmaceutical Co., Ltd, Nanjing, China, 0.5 g/tablet, Approval No.: SFDA Approval Number H20030087), do not mention fever as an adverse effect in their instructions. Additionally, most clinicians are not aware of this particular side effect of this drug. As a result, the cause of fever is not promptly identified upon admission, and patients often receive empirical anti-infective therapy, resulting in recurrent fever. The mechanism of calcium dobesilate-induced drug fever remains unclear, and the patient’s constitution may be a cause.

### 3.1. Strengths and limitations

#### 3.1.1. Strengths.

The patient took calcium dobesilate, which caused high fever. After stopping calcium dobesilate, his body temperature returned to normal. The diagnosis of calcium dobesilate-induced hyperpyrexia is clear, and the treatment is effective.

#### 3.1.2. Limation.

First, clinical reports on calcium dobesilate-induced hyperpyrexia are relatively rare, and large-scale, multicenter clinical studies are still needed to further verify whether calcium dobesilate can cause fever. Second, calcium dobesilate-induced hyperpyrexia has no clinical specificity and is more likely to be misdiagnosed as infection-induced fever, so anti-infection treatment should be given, which delays the treatment and diagnosis of this disease.

## 4. Conclusion

Briefly, the patient’s body temperature should be monitored during the use of calcium dobesilate in clinical therapy. In cases of unexplained hyperpyrexia, calcium dobesilate-induced fever should be considered a diagnosis. In such a case, calcium dobesilate should be discontinued promptly to ensure patient safety and clinical treatment efficacy.

## Acknowledgments

This work was supported by the Nature Science Foundation of China under Grant No. 81960350 and the Yunnan Applied Basic Research Project-Union Foundation of China under Grant No. 202201AY070001-091.

## Author contributions

**Conceptualization:** Hui Yang, Hong-Ling Yuan, Hong-Kui Zhang.

**Data curation:** Zhi-Ping Zhang, Hong-Kui Zhang, Ming-Wei Liu.

**Formal analysis:** Hui Yang, Hong-Ling Yuan.

**Funding acquisition:** Zhi-Ping Zhang, Ming-Wei Liu.

**Investigation:** Hui Yang, Hong-Ling Yuan.

**Methodology:** Hong-Ling Yuan.

**Project administration:** Zhi-Ping Zhang.

**Resources:** Ming-Wei Liu.

**Software:** Zhi-Ping Zhang.

**Supervision:** Hui Yang, Hong-Kui Zhang, Ming-Wei Liu.

**Visualization:** Zhi-Ping Zhang.

**Writing – original draft:** Hui Yang, Hong-Kui Zhang.

**Writing – review & editing:** Ming-Wei Liu.
